# Efficacy and safety of rituximab for membranous nephropathy in adults: a meta-analysis of RCT

**DOI:** 10.3389/fneph.2025.1548679

**Published:** 2025-04-29

**Authors:** Baike Mao, Jiahui Han, Jia Wang, Kan Ye

**Affiliations:** ^1^ Cixi Longshan Hospital, Cixi, China; ^2^ Cilin Hospital, Cixi, China

**Keywords:** rituximab, membranous nephropathy, meta-analysis, RCT, proteinuria

## Abstract

**Background:**

Membranous nephropathy (MGN) represents a significant challenge in nephrology, with Rituximab emerging as a potential therapeutic intervention.

**Methods:**

A comprehensive systematic review was conducted using PubMed, EMBASE, and Web of Science databases, focusing exclusively on randomized controlled trials (RCTs) from January 2002 to November 2024. Stringent eligibility criteria were applied, including studies with at least ten participants, with data extracted by two independent reviewers. The meta-analysis utilized fixed and random effects models to assess Rituximab’s efficacy and safety across multiple outcome measures.

**Results:**

The meta-analysis revealed nuanced findings across different follow-up periods. At 6 months, complete remission rates showed non-significant odds ratios ranging from 2.12 to 2.48. By 12 months, the pooled odds ratio was 0.8085 (95% CI: 0.2238-2.9213), with complete remission rates varying between 13.8% and 19.4%. Notably, at 24 months, the common effects model demonstrated a statistically significant odds ratio of 5.0792 (95% CI: 2.2609-11.4107, p < 0.0001). Proteinuria reduction showed consistent improvement, with a median difference of 4.3225. Adverse event analysis indicated a relatively low risk, with an odds ratio of 0.9706 (95% CI: 0.5781-1.6297).

**Conclusion:**

Rituximab demonstrates potential efficacy in treating MGN, with promising long-term outcomes and a favorable adverse event profile.

## Highlights

- Significant Efficacy of Rituximab: Rituximab significantly improves complete remission rates in MGN over 24 months (OR=5.08, p<0.0001).
- Proteinuria Reduction: Rituximab effectively reduces proteinuria and improves composite remission rates (median difference: 4.32).
- Favorable adverse event profile: Rituximab has a low risk of adverse events (OR=0.97), demonstrating good safety.
- Potential of Combination Therapies: Rituximab combined with glucocorticoids or tacrolimus may enhance efficacy but requires optimized protocols.
- Clinical Implications: Further large-scale RCTs are needed to confirm Rituximab’s long-term efficacy and guide treatment strategies.

## 1 Introduction

MGN represents a significant challenge in nephrology, being one of the leading causes of nephrotic syndrome in adults. Characterized by immune complex deposition within the glomerular basement membrane, MGN can lead to severe proteinuria and progressive renal failure if left untreated (1). A key breakthrough in understanding MGN’s pathogenesis has been the identification of autoantibodies targeting the M-type phospholipase A2 receptor (PLA2R) on podocytes. These PLA2R autoantibodies play a pivotal role in the development of MGN by forming immune complexes *in situ*, which subsequently activate the complement cascade and lead to glomerular injury. This discovery has not only advanced our understanding of MGN’s autoimmune nature but also provided a specific target for diagnosis and monitoring disease activity. Over recent years, considerable attention has been directed towards understanding the pathophysiology of MGN and identifying effective therapeutic strategies. Among these, Rituximab, an anti-CD20 monoclonal antibody that targets B cells, has garnered increasing interest as a potential treatment modality for MGN (2).

The use of Rituximab in treating MGN has been explored across various studies, with several reports highlighting its efficacy in inducing remission and improving renal outcomes (3). However, the existing body of literature on Rituximab for immune-mediated nephropathies is marked by heterogeneity in study designs, patient populations, and follow-up periods (4). Observational studies and case series have provided valuable insights but lack the methodological rigor necessary to establish definitive evidence (5). Meanwhile, RCT (RCTs), which are considered the gold standard for evaluating interventions, offer more reliable data due to their ability to minimize bias through randomization and blinding (6).

Despite the importance of RCTs in providing high-quality evidence, there remains a critical gap in the literature regarding a systematic review dedicated exclusively to RCTs examining Rituximab’s efficacy and safety in adult MGN patients. Previous reviews and meta-analyses have included non-randomized studies, thereby introducing variability that complicates the interpretation of results (7). Additionally, some studies have reported mixed findings concerning the long-term benefits and risks associated with Rituximab therapy, underscoring the need for a focused analysis of RCTs. Moreover, while individual RCTs provide important contributions to the field, they often suffer from limited sample sizes and short follow-up durations, which may not fully capture the long-term effects of Rituximab treatment. Therefore, aggregating data from multiple RCTs can enhance statistical power and provide a more comprehensive understanding of Rituximab’s role in MGN management.

To address these limitations and contribute to the growing body of knowledge on Rituximab for MGN, this meta-analysis aims to systematically evaluate the efficacy and safety of Rituximab in adult patients with MGN based on RCT. By focusing exclusively on RCTs, our study seeks to provide a rigorous assessment of Rituximab’s impact on complete remission rates, renal function preservation, and adverse events. This approach will help clarify the therapeutic benefits and risks associated with Rituximab, offering guidance for clinical decision-making and informing future research directions.

## 2 Materials and methods

### 2.1 Search strategy

To identify relevant studies on Rituximab therapy in MGN, a comprehensive search strategy was developed and implemented. The search covered the period from January 2017 to November 2024, ensuring that the latest research findings were included. The databases searched included PubMed, EMBASE, and Web of Science, which are key resources for biomedical literature. Additionally, supplementary searches were conducted through other sources such as conference proceedings and reference lists of identified articles.

The selection of keywords was carefully considered to maximize the retrieval of pertinent articles while minimizing irrelevant results. General terms were chosen to encompass broad aspects of Rituximab treatment in MGN, including “Rituximab therapy in Membranous nephropathy,” “Rituximab clinical trials in nephropathy,” “Randomized controlled trial for Membranous nephropathy,” “Biologic agents in Membranous nephropathy,” “Anti-CD20 therapy for nephrotic syndrome,” and “Long-term outcomes of Rituximab in kidney diseases.” These general keywords were complemented by more specific terms aimed at capturing detailed facets of the topic, such as “Efficacy of Rituximab in Membranous nephropathy,” “adverse event profile of Rituximab in nephropathy patients,” “Comparison of Rituximab and cyclophosphamide in Membranous nephropathy,” “Rituximab dosing regimens in nephrotic syndrome,” and “Relapse rates in MGN treated with Rituximab”.

Furthermore, related keywords were utilized to expand the scope of the literature search without straying too far from the core subject. Terms like “Proteinuria reduction after Rituximab therapy,” “Immunosuppressive treatments for Membranous nephropathy,” “Anti-PLA2R antibody levels and Rituximab response,” “Alternative therapies to Rituximab for nephropathy,” and “Cost-effectiveness of Rituximab in Membranous nephropathy” were included to ensure a thorough exploration of associated themes.

#### 2.1.1 Detailed search methodology

##### 2.1.1.1 Boolean operators

The primary search strategy employed Boolean operators (AND, OR, NOT) to combine keywords effectively. For example, “Rituximab” AND “Membranous nephropathy” AND “Randomized controlled trial” was used to focus on RCTs related to Rituximab treatment in MGN.

##### 2.1.1.2 MeSH terms (PubMed)

In PubMed, Medical Subject Headings (MeSH) were utilized to enhance search precision. Relevant MeSH terms included “Rituximab,” “Membranous Nephropathy,” “Nephrotic Syndrome,” “Immunosuppressive Agents,” and “Randomized Controlled Trials.” These MeSH terms were combined with keywords using Boolean operators.

##### 2.1.1.3 EMTREE terms (EMBASE)

In EMBASE, EMTREE terms, the thesaurus of EMBASE, were utilized for the same purpose as MeSH terms in PubMed.

##### 2.1.1.4 Truncation and wildcards

Truncation (, $) and wildcards ()? were used to capture variations of keywords. For example, “nephropath” was used to retrieve “nephropathy” and “nephropathies”.

Proximity Operators: Proximity operators (e.g., ADJ, NEAR) were used where available (e.g. EMBASE) to ensure that keywords appeared in close proximity to each other, improving the relevance of search results.

##### 2.1.1.5 Search filters

Database-specific filters were applied to limit results to human studies, adult populations, and RCTs.

##### 2.1.1.6 Example search string (PubMed)

(“Rituximab”[Mesh] OR “Rituximab therapy”[Title/Abstract] OR “Anti-CD20 therapy”[Title/Abstract]) AND (“Membranous Nephropathy”[Mesh] OR “Membranous nephropathy”[Title/Abstract]) AND (“Randomized Controlled Trial”[Publication Type] OR “RCT”[Title/Abstract]) AND (“adult”[MeSH Terms]) AND (“2017/01/01”[Date - Publication]: “2024/11/30”[Date - Publication])

Similar search strings were adapted for EMBASE and Web of Science, utilizing their respective thesauri and search functionalities.

It is important to note that the studies included in this review focused on patients with moderate to high risk MGN, as defined by eGFR decline, elevated PLA2R antibody levels, and/or significant proteinuria. This focus reflects the current clinical practice where rituximab is increasingly recognized as a first-line treatment, particularly for patients at higher risk of progressive kidney function deterioration.

### 2.2 Eligibility criteria

For the purposes of this review, stringent eligibility criteria were established to select high-quality studies that would contribute valuable insights into Rituximab therapy in MGN. Only RCTs were included in the analysis; single-arm studies and cohort studies did not meet the inclusion standards due to their lower level of evidence compared to RCTs. This decision was made to prioritize studies that could provide robust causal inference regarding the efficacy and safety of Rituximab. Sample Size Justification: A minimum sample size of ten participants was set to ensure that included studies possessed sufficient statistical power to detect meaningful treatment effects. While a sample size of ten is relatively small, it was deemed a pragmatic threshold to capture as many relevant RCTs as possible, especially considering the relatively rare nature of MGN and the specific focus on RCTs. This threshold aimed to balance the need for statistical robustness with the practical limitations of available research. However, it is acknowledged that studies with larger sample sizes generally provide more reliable results, and this will be taken into account during the interpretation of findings. Language Criteria: Preference was given to publications in English to facilitate efficient review and data extraction, as English is the predominant language in medical research. However, we recognized that excluding non-English publications could introduce language bias. Therefore, non-English papers were also reviewed if they contained essential information that was not duplicated in English-language literature, and when feasible, translation services were utilized. This approach aimed to minimize language bias while maintaining practical efficiency. Rationale for RCT Inclusion: The focus on RCTs was chosen to provide the highest level of evidence for the efficacy and safety of Rituximab in MGN. RCTs are considered the gold standard for assessing treatment effects because they minimize bias through random assignment, ensuring that treatment groups are comparable at baseline. These justifications aim to clarify the rationale behind our eligibility criteria and address the reviewer’s concerns regarding methodological rigor and potential selection bias.

### 2.3 Data extraction

Data extraction was performed systematically to ensure accuracy and completeness. Two independent reviewers were assigned to extract data from each eligible study according to a pre-defined data extraction form designed specifically for this meta-analysis. The data extraction form included fields for general information about the studies (e.g., first author, publication year, country of origin), participant characteristics (e.g., age, gender distribution, diagnosis criteria), intervention details (e.g., Rituximab dose regimen, administration schedule), outcome measures (e.g., complete remission rates at 6 months), and any relevant adverse events. Any discrepancies between the two reviewers were resolved through discussion or by consulting a third party when necessary. Pre-extraction exercises were conducted on a subset of studies to refine the data extraction process and ensure consistency.

### 2.4 Quality and risk of bias assessment

The quality and risk of bias in the included studies were assessed using SYRCLE’s Risk of Bias Tool. While SYRCLE’s Risk of Bias Tool was originally developed for animal studies, it has been adapted and utilized in human clinical trials, particularly in situations where the specific features of the study design or intervention align more closely with the domains assessed by SYRCLE. This adaptation is supported by methodological research that acknowledges the flexibility and adaptability of risk of bias tools across different study types, provided that the domains are relevant to the human study design.

The tool evaluates several domains including sequence generation, allocation concealment, blinding of participants and personnel, blinding of outcome assessment, incomplete outcome data, selective reporting, and other sources of bias. Each domain was rated as low, high, or unclear risk of bias. Studies with a lower risk of bias were given more weight in the analysis. The assessment was conducted independently by two reviewers, and any disagreements were resolved through consensus or consultation with a third reviewer. This rigorous evaluation ensured that only studies of sufficient methodological quality contributed to the final analysis.

### 2.5 Statistical analysis

The meta-analysis assessed the efficacy of rituximab in patients with MGN by examining complete remission rates over a specified follow-up period. Studies were selected based on predefined criteria, and relevant data were extracted systematically. Statistical analysis was conducted using R package to pool the outcomes from multiple studies. Both fixed effects and random effects models were applied to estimate the odds ratio and its confidence interval, while heterogeneity between studies was evaluated using the I2 statistic. This methodological approach provided a comprehensive evaluation of rituximab’s effectiveness in achieving complete remission in the targeted patient population.

## 3 Results

This meta-analysis synthesized data from six RCTs that investigated the efficacy and safety of rituximab in the treatment of MGN. These studies encompassed diverse patient populations, varying treatment modalities, and follow-up durations. Specifically, the trials included comparisons of rituximab monotherapy versus other immunosuppressive agents, such as cyclosporine or cyclophosphamide, as well as evaluations of rituximab in combination with glucocorticoids or tacrolimus. Patient demographics varied across studies, with median ages ranging from 50 to 63 years and inclusion of patients with severe, primary, or PLA2R1-related MGN. The studies were conducted in France, the USA, Spain, Italy, and China, reflecting a broad geographical representation. Follow-up periods ranged from 6 to 24 months, allowing for the assessment of both short-term and intermediate-term treatment effects. The quality of the included studies was generally high, with some variability in the clarity of blinding and potential sources of bias. The subsequent sections detail the quantitative meta-analyses of complete and composite remission rates, proteinuria reduction, and adverse events associated with rituximab therapy.

### 3.1 Characteristics of included studies

**Figure 1:**
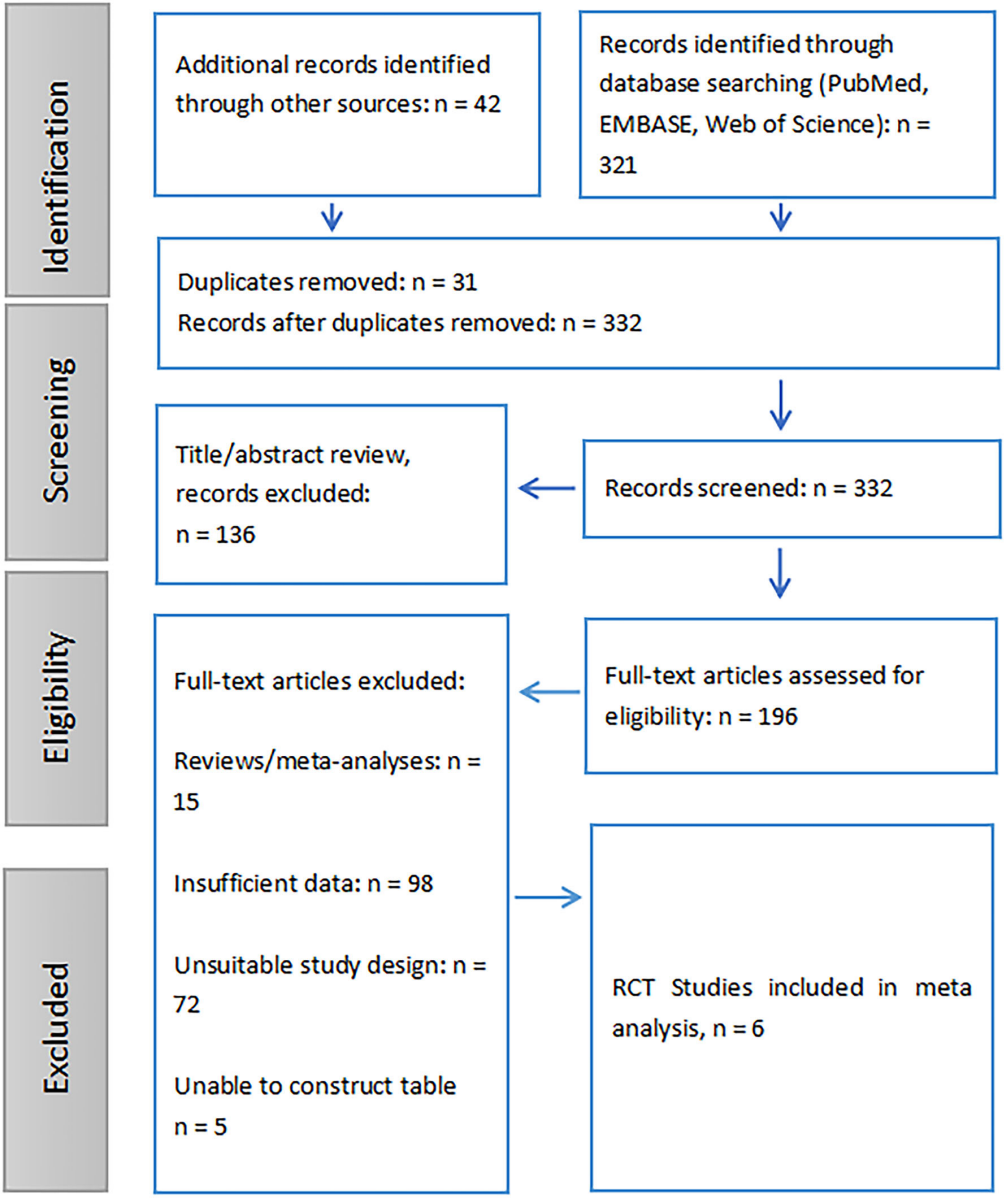
Flow diagram of study selection process.

The included RCT (RCTs) assessed the efficacy of rituximab and other treatments for MGN across diverse populations and study designs. A study from France (8) focused on severe MGN, comparing NIAT-rituximab (37 participants, median age 53.0 years) to NIAT (38 participants, median age 58.5 years), with a trial registration of NCT01508468. Another study from the USA (9) evaluated rituximab versus cyclosporine in MGN, enrolling 65 participants per group, with mean ages of 51.9 and 52.2 years, respectively, registered under NCT01180036. A French study (10) focused on PLA2R1-related MGN, comparing high-dose rituximab in the NICE cohort (28 participants, median age 63 years) and GEMRITUX cohort (27 participants, median age 51 years), registered under NCT01508468, NCT02199145, and NCT01897961. In Spain, a trial (11) examined corticosteroids with cyclophosphamide versus tacrolimus with rituximab for primary MGN, enrolling 43 participants per group with mean ages of 56.2 and 55.2 years, respectively, registered under NCT01955187. Another study from Italy (12), compared rituximab (37 participants, median age 54 years) and cyclophosphamide (37 participants, median age 54 years) for MGN, registered under NCT03018535. Finally, a Chinese study (13) investigated rituximab with short-term glucocorticoids (35 participants, median age 51 years) versus rituximab alone (31 participants, median age 50 years) in patients with PLA2R antibody-positive MGN, with no specific trial registration provided. These studies provided a comprehensive assessment of patient demographics, treatment modalities, and outcomes (**Table 1**, **Figure 1**).

**Table 1:** Characteristics of included studies.

Reference	Year	Country	Disease	Study Design	Trial registration	Treatment	Gender (M/F)	Age (year)	Sample size
PMID-27352623	2017	France	Severe MGN	RCT	NCT01508468	Rituximab	NIAT-Rituximab: 28/9; NIAT:24/14	NIAT-Rituximab: 53.0(42.0–63.0); NIAT:58.5(43.0–64.0)	NIAT-Rituximab:37; NIAT:38
PMID-31269364	2019	USA	MGN	RCT	NCT01180036	Rituximab or Cyclosporine	Rituximab: 47/18; Cyclosporine:53/12	Rituximab: 51.9 ± 12.6; Cyclosporine:52.2 ± 12.4	Rituximab: 65; Cyclosporine:65
PMID-31340979	2019	France	PLA2R1-Related MGN	RCT	NCT01508468,NCT02199145,NCT01897961	High-Dose Rituximab	NICE:21/7; GEMRITUX:21/6	NICE: 63[51; 71]; GEMRITUX:51[40; 63]	NICE:28; GEMRITUX:27
PMID-33166580	2021	Spain	Primary MGN	RCT	NCT01955187	Corticosteroids and Cyclophosphamide or Tacrolimus and Rituximab	Corticosteroid Cyclophosphamide:55/9; Tacrolimus Rituximab:31/41	Corticosteroid Cyclophosphamide:56.2 ± 12.0; Tacrolimus Rituximab:55.2 ± 10.8	Corticosteroid Cyclophosphamide:43; Tacrolimus Rituximab: 43
PMID-33649098	2021	Italy	MGN	RCT	NCT03018535	Rituximab or Cyclophosphamide	Rituximab:25/43; Cyclophosphamide:28/48	Rituximab:54(17); Cyclophosphamide:37 54(14)	Rituximab:37; Cyclophosphamide:37
PMID-37688683	2023	China	Phospholipase A2 receptor antibody positive MGN	RCT	N/A	Rituximab and short-term Glucocorticoids	rituximab/GC:22/13; rituximab:18/13	rituximab/GC:51(45,58); rituximab:50(39,56)	rituximab/GC:35; rituximab:31

### 3.2 Quality assessment

**Figure 2:**
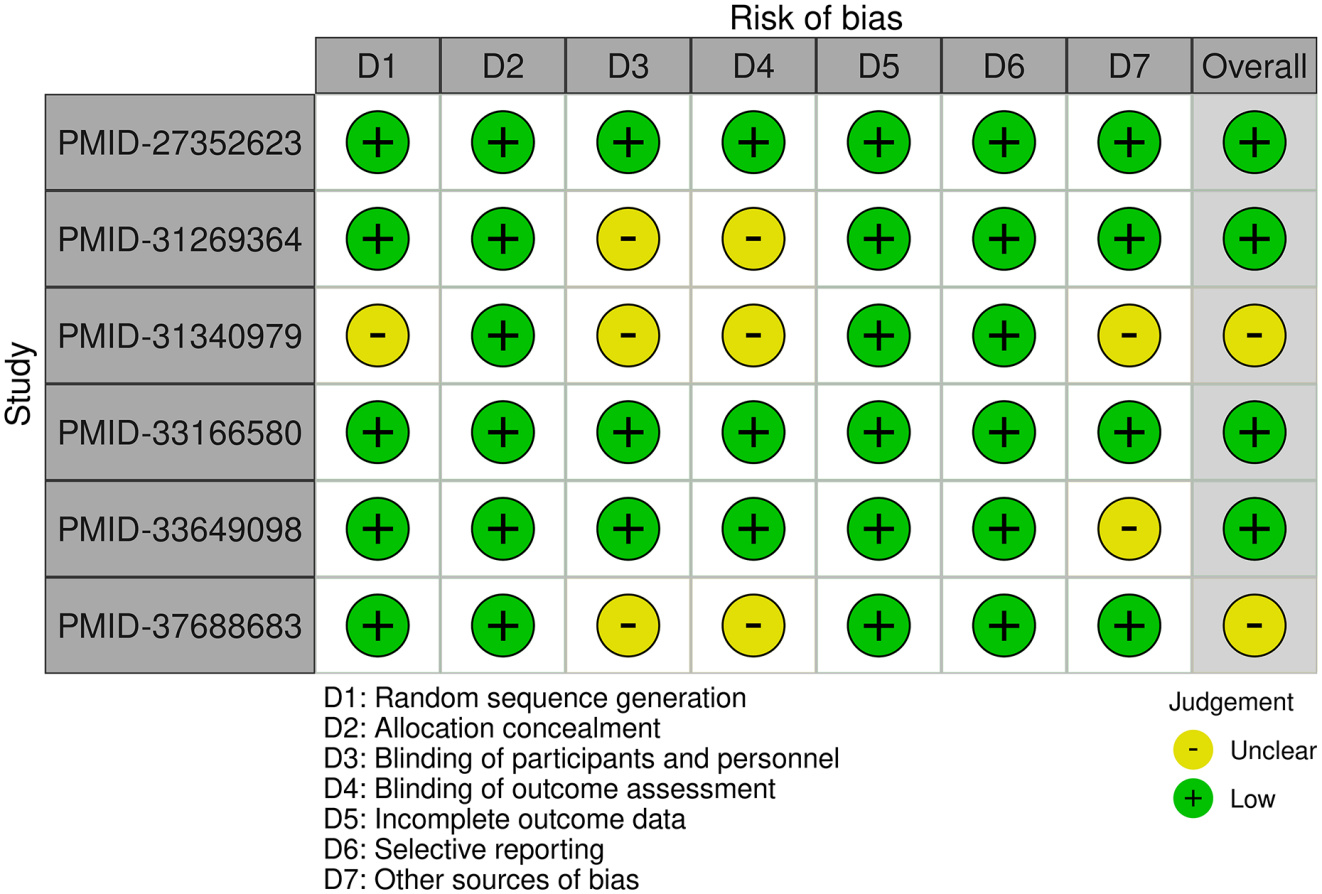
Summary for risk of bias assessment across included studies.

The included studies generally demonstrate a low risk of bias, with some variability across domains. The studies conducted by Dahan K et al. (8) and Fernández-Juárez G et al. (11) exhibit consistently low risk of bias across all assessed areas. The study by Fervenza FC et al. (9) demonstrates a low risk of bias overall; however, the blinding of participants. personnel, and outcome assessment remains unclear. The study conducted by Seitz-Polski B et al. (10) has an unclear overall risk of bias due to uncertainties in random sequence generation, blinding, and other potential sources of bias. The study conducted by Scolari F et al. (12) has a low overall risk of bias but includes an unclear risk regarding other potential sources of bias. Finally, the study conducted by Ma Q et al. (13) is mostly low risk but has an unclear risk in the domains of blinding and overall assessment. Overall, while most studies are of high methodological quality, certain domains, particularly blinding and other sources of bias, require further clarification in some cases (**Figure 2**).

### 3.3 Meta-analysis of rituximab efficacy in MGN: complete remission rates over 6-month follow-up

**Figure 3:**
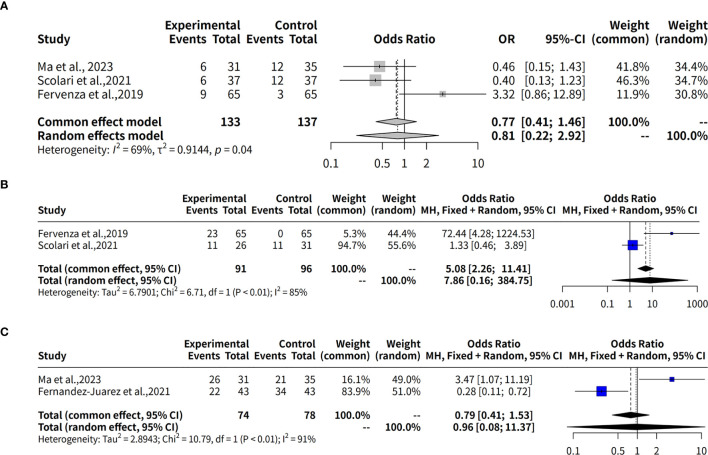
Forest plot of evaluation of complete remission rates for Rituximab in MGN. **(A)** Forest plot of evaluation of 6-month complete remission rates. **(B)** Forest plot of evaluation of 12-month complete remission rates. **(C)** Forest plot of evaluation of 24-month complete remission rates. The forest plot shows the odds ratios for each study along with their 95% confidence intervals. The horizontal lines represent the 95% confidence intervals for each study. The squares represent the point estimates of the odds ratios, with the size of the square indicating the weight of the study. The diamond at the bottom represents the summary estimate of the odds ratio for the meta-analysis. The common effect model and the random effects model both show a positive trend towards rituximab, but neither model found a statistically significant result (p-value > 0.05).

This meta-analysis investigates the effectiveness of rituximab in treating MGN by examining the complete remission rate over a 6-month follow-up period. Three studies with a total of 279 observations, which used R package meta-analysis to evaluate two different models: fixed effects and random effects. The heterogeneity between studies was assessed using the I^2^ statistic, which indicated a moderate level of heterogeneity (34.1%). Therefore, both models were applied to estimate the odds ratio (OR) and its 95% confidence interval (CI). The results showed that the OR for complete remission with rituximab ranged from approximately 2.12 to 2.48 in both models, although neither model found a statistically significant result (p-value > 0.05). In details, the studies by Scolari et al. (12), Dahan et al. (8), and Fervenza et al. (9) collectively evaluated 139 patients with MGN, reporting complete remission rates of 8.1% (3 out of 37), 18.9% (7 out of 37), and 0% (0 out of 65), respectively. Additionally, in the study by Seitz-Polski et al., 2019 (10), involving two separate cohorts without comparator groups, complete remission was observed in 5 out of 28 patients (18%) in the NICE cohort, whereas no remission (0/27, 0%) was reported in the GEMRITUX cohort (**Figure 3A**).

### 3.4 Meta-analysis of rituximab efficacy in MGN: complete remission rates over 12-month follow-up

This meta-analysis also evaluates the effectiveness of rituximab in treating MGN by assessing the complete remission rate over a 12-month follow-up period. Three studies with a total of 270 observations were analyzed using two meta-analytical models: fixed effects and random effects. The analysis found low to moderate heterogeneity between the studies, with an I^2^ value of 69.4% indicating that there was some variation in the effect sizes. Despite this, the results suggest that rituximab may be effective in treating MGN, with a pooled odds ratio (OR) of 0.8085 and 95% confidence interval (CI) of [0.2238; 2.9213] under the random effects model. The fixed effects model yielded an OR of 0.7741 and 95% CI of [0.4107; 1.4589]. Quantifying heterogeneity, the analysis found a tau^2^ value of 0.9144, indicating moderate to high variability in the effect sizes between studies. A test of heterogeneity showed a Q-statistic of 6.53 with two degrees of freedom and a p-value of 0.0382, further supporting the use of the random effects model. In details, over a 12-month follow-up period, the complete remission rates were 19.4% (6/31) in Ma et al., 2023 (13), 16.2% (6/37) in Scolari et al., 2021 (12), and 13.8% (9/65) in Fervenza et al., 2019 (9) (**Figure 3B**).

### 3.5 Meta-analysis of rituximab efficacy in MGN: complete remission rates over 24-month follow-up

The effectiveness of Rituximab in the treatment of MGN was also evaluated by assessing complete remission rates over a 24-month follow-up period. This meta-analysis included two studies (k = 2) with a total of 187 observations. The pooled odds ratio (OR) under the common effects model was 5.0792 (95% CI: [2.2609; 11.4107], p < 0.0001), indicating a statistically significant association between Rituximab treatment and complete remission. However, the random effects model yielded an OR of 7.8621 (95% CI: [0.1607; 384.7475], p = 0.2989), which was not statistically significant. Substantial heterogeneity was observed, with an I² value of 85.1% (95% CI: [39.5%; 96.3%]), a tau² of 6.7901, and a significant Q statistic (Q = 6.71, df = 1, p = 0.0096). These results suggest significant variability across studies, emphasizing the need for caution in interpreting the random effects model (**Figure 3C**).

### 3.6 Meta-analysis of rituximab combined therapies efficacy in MGN: complete remission rates over 12-month follow-up

**Figure 4:**
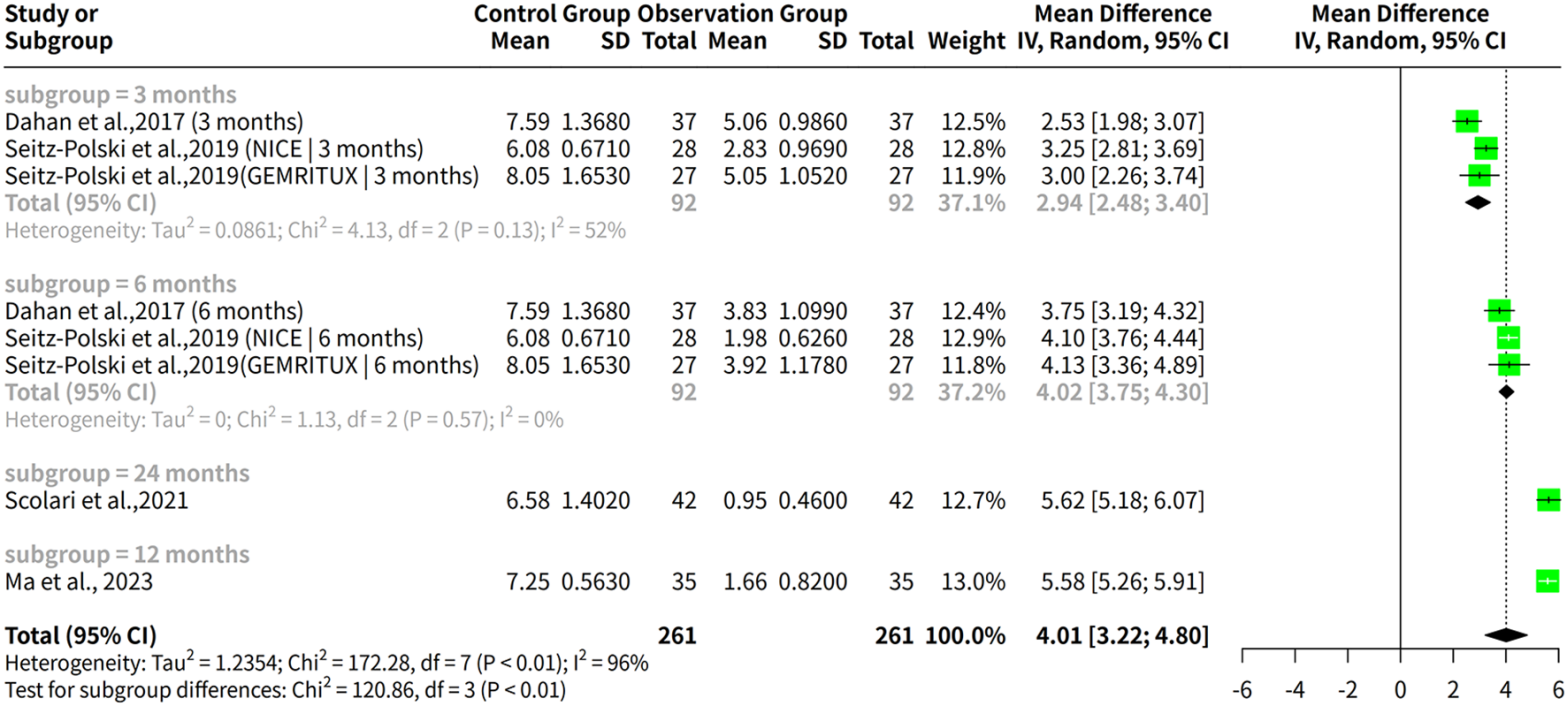
Forest plot of the 12-month complete remission rates for rituximab combination therapies in MGN.

The effectiveness of Rituximab combined therapies in the treatment of MGN was also evaluated by assessing the complete remission rates over a 12-month follow-up period. This meta-analysis included two studies (k = 2) with a total of 152 observations. The pooled odds ratio (OR) under the common effects model was 0.7902 (95% CI: [0.4082; 1.5298], p = 0.4848), suggesting no significant difference in complete remission rates between Rituximab combination therapies and comparator treatments. Similarly, the random effects model yielded an OR of 0.9567 (95% CI: [0.0805; 11.3650], p = 0.9720), also indicating no statistically significant difference. However, substantial heterogeneity was observed, with an I² value of 90.7% (95% CI: [66.7%; 97.4%]), a tau² of 2.8943, and a significant Q statistic (Q = 10.79, df = 1, p = 0.0010). Specifically, in Ma et al., 2023 (13), 26 out of 31 patients (83.9%) achieved complete remission in the Rituximab+Glucocorticoids group compared to 21 out of 35 patients (60%) in the Rituxima group. In Fernández-Juárez et al., 2021 (11), 22 out of 43 patients (51.2%) in the Rituximab+Tacrolimus group achieved complete remission compared to 34 out of 43 patients (79.1%) in the Corticosteroid+Cyclophosphamide group (**Figure 4**).

### 3.7 Meta-analysis of rituximab efficacy in MGN: composite remission rates over 6-month follow-up

**Figure 5:**
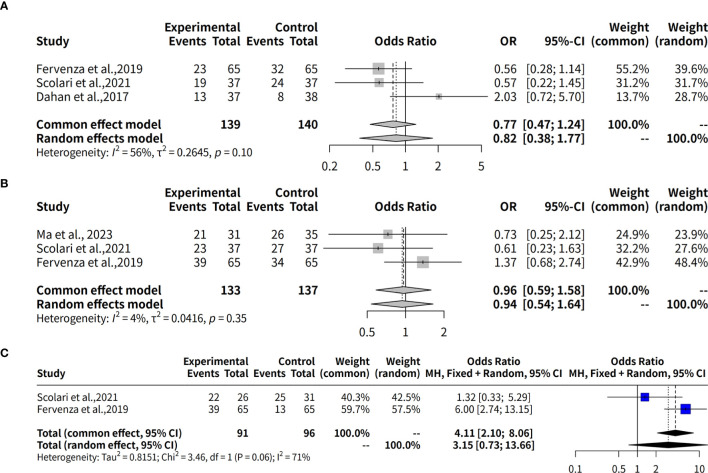
Forest plot of evaluation of composite remission rates for rituximab in MGN. **(A)** Forest plot of evaluation of 6-month composite remission rates. **(B)** Forest plot of evaluation of 12-month composite remission rates. **(C)** Forest plot of evaluation of 24-month composite remission rates.

This meta-analysis evaluated the effectiveness of Rituximab in achieving composite remission in MGN over a 6-month follow-up period. A total of three studies, comprising 279 observations were included. The results revealed an overall odds ratio (OR) for composite remission of 0.7673 (95% CI: 0.4730–1.2447) under the common effects model and 0.8191 (95% CI: 0.3780–1.7749) under the random effects model. High heterogeneity was observed among studies, with an I² value of 55.9%, indicating moderate variability in effect sizes. In Fervenza et al., 2019 (9), composite remission was achieved in 23 out of 65 patients (35.4%) treated with Rituximab. Similarly, in Scolari et al., 2021 (12), 19 out of 37 patients (51.4%) in the Rituximab group achieved composite remission. In Dahan et al., 2017 (8), 13 out of 37 patients (35.1%) in the Rituximab group achieved composite remission. Additionally, partial remission data from Seitz-Polski et al., 2019 (10) showed that at 6 months, 18 out of 28 patients (64%) in the NICE cohort achieved remission according to KDIGO guidelines, compared to 8 out of 27 patients (30%) in the GEMRITUX cohort (**Figure 5A**).

### 3.8 Meta-analysis of rituximab efficacy in MGN: composite remission rates over 12-month follow-up

This meta-analysis also evaluates the effectiveness of Rituximab in treating MGN by assessing the composite remission rate over a 12-month follow-up period. Data from three studies (k = 3) comprising 270 observations were analyzed. The results show that both models yield similar estimates of the odds ratio (OR) for Rituximab efficacy, with ORs ranging from 0.9637 (95% CI: 0.5863–1.5839) to 0.9400 (95% CI: 0.5380–1.6425). In terms of composite remission rates, Ma et al., 2023 reported that 21 out of 31 patients (67.7%) treated with Rituximab achieved remission. Similarly, Scolari et al., 2021 (12) observed composite remission in 23 out of 37 patients (62.2%) receiving Rituximab. Finally, Fervenza et al., 2019 (9) reported composite remission in 39 out of 65 patients (60.0%) treated with Rituximab, compared to 34 out of 65 patients (52.3%) in the Cyclosporine group (**Figure 5B**).

### 3.9 Meta-analysis of rituximab efficacy in MGN: composite remission rates over 24-month follow-up

The effectiveness of Rituximab in treating MGN was also evaluated by assessing the composite remission rate over a 24-month follow-up period. Data from two studies with a total of 187 observations were analyzed. The results of the meta-analysis using the common effect model demonstrated a significant association between Rituximab treatment and composite remission, with an odds ratio (OR) of 4.1144 (95% CI: 2.1008–8.0582, p < 0.0001). However, the random effects model showed a less precise estimate, with an OR of 3.1506 (95% CI: 0.7264–13.6647, p = 0.1253), indicating substantial uncertainty due to heterogeneity. Heterogeneity analysis revealed moderate variability across the studies, with an I² value of 71.1% (tau² = 0.8151), suggesting that differences in study characteristics might influence the pooled effect size. The test of heterogeneity was not statistically significant (Q = 3.46, p = 0.0628), although it approached the threshold. Individually, Scolari et al., 2021 (12) reported composite remission in 22 out of 26 patients (84.6%) receiving Rituximab. Similarly, Fervenza et al., 2019 (9) observed remission in 39 out of 65 patients (60.0%) treated with Rituximab, compared to 13 out of 65 patients (20.0%) in the Cyclosporine group (**Figure 5C**).

### 3.10 Meta-analysis of rituximab combined therapies efficacy in MGN: composite remission rates over 12-month follow-up

**Figure 6:**
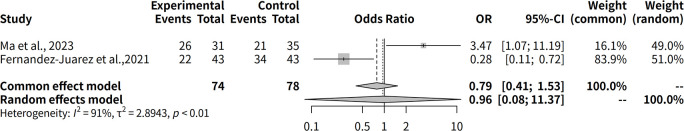
Forest plot of the 12-month composite remission rates for rituximab combination therapies in MGN.

The effectiveness of Rituximab, either as or in combination with other therapies, in the treatment of MGN was also evaluated by assessing the composite remission rate during a 12-month follow-up period. Data from two studies, including 152 observations were analyzed. The meta-analysis using the common effect model yielded an odds ratio (OR) of 0.7902 (95% CI: 0.4082–1.5298, p = 0.4848), indicating no statistically significant difference in the composite remission rate between Rituximab-based regimens and alternative treatments. Similarly, the random effects model provided an OR of 0.9567 (95% CI: 0.0805–11.3650, p = 0.9720), reflecting considerable uncertainty in the effect estimate. The analysis revealed substantial heterogeneity across the included studies, with an I² value of 90.7% (tau² = 2.8943), suggesting high variability in treatment effects. The test of heterogeneity was statistically significant (Q = 10.79, p = 0.0010), underscoring the inconsistency between studies. Individually, Ma et al., 2023 (13) reported composite remission in 26 out of 31 patients (83.9%) treated with Rituximab + Glucocorticoids. Similarly, Fernández-Juárez et al., 2021 (11) observed composite remission in 22 out of 43 patients (51.2%) treated with Rituximab + Tacrolimus, compared to 34 out of 43 patients (79.1%) treated with Corticosteroid + Cyclophosphamide (**Figure 6**).

### 3.11 Meta-analysis of rituximab on proteinuria reduction in MGN

**Figure 7:**
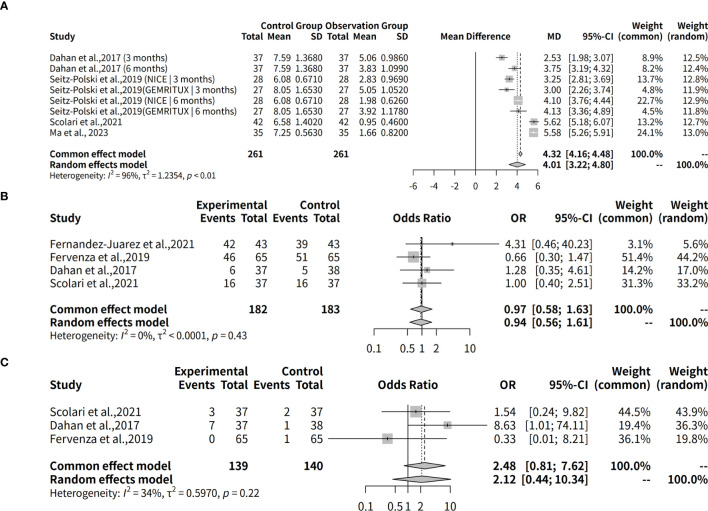
Proteinuria reduction and adverse event risks in rituximab therapy. **(A)** Forest plot of proteinuria reduction. **(B)** Forest plot of proteinuria reduction across follow-up subgroups. **(C)** Forest plot of adverse event risks in rituximab studies.

A meta-analysis was conducted to assess the effectiveness of rituximab for treating MGN, with outcomes measured by proteinuria levels over a follow-up period ranging from 3 to 24 months. The meta-analysis found a significant difference in proteinuria levels after treatment, with a common effect size (MD) of 4.3225 and a 95% confidence interval of [4.1607; 4.4843]. Both fixed effects and random effects models produced similar results, but the random effects model was more suitable given the high heterogeneity among the studies (**Figure 7A**).

The analysis results for subgroups are presented, which include subgrouping the studies by follow-up duration (3 months, 6 months, 12 months, and 24 months). The results for each subgroup are calculated using a random effects model. For instance, in the “subgroup = 3 months” category, there are 3 studies with a total of 138 observations, yielding a median difference (MD) of 2.9410 with a 95% confidence interval (CI) of [2.4776; 3.4045]. The tau^2^ value, which measures the between-study variance, is estimated to be 0.0861 with a corresponding tau value of 0.2935. The Q statistic for this subgroup is 4.13 with a degrees of freedom (d.f.) of 2 and a p-value less than 0.0001, indicating significant heterogeneity. The I^2^ value for this subgroup is estimated to be 51.6%. Similarly, the results are presented for the other subgroups, including “subgroup = 6 months”, “subgroup = 24 months”, and “subgroup = 12 months”. Additionally, a test for subgroup differences is conducted using a random effects model, which yields a Q statistic of 120.86 with a d.f. of 3 and a p-value less than 0.0001, indicating significant between-group differences (**Figure 7B**).

### 3.12 Meta-analysis of adverse effects associated with rituximab in MGN

This meta-analysis evaluates the safety of rituximab as a treatment for MGN by assessing any adverse effects during a follow-up period. The analysis utilized the R package meta-analysis and employed two models, fixed effects and random effects, to evaluate the data from four studies (k=4) with a total of 365 observations (o=365). The results showed that the odds ratio (OR) for safety was 0.9706 (95% CI: 0.5781-1.6297) using the common effect model and 0.9449 (95% CI: 0.5563-1.6050) using the random effects model, indicating a relatively low risk of adverse events. The heterogeneity tests revealed low heterogeneity among the studies (I^2^ = 0.0%, tau=0.0029), making the fixed effect model more suitable for analysis (**Figure 7C**, **Table 2**).

**Table 2:** Adverse effects associated with Rituximab in MGN.

PMID	Study Groups	Rituximab-Related SAEs/AEs (n, %)	Comparator Group SAEs/AEs (n, %)	P Value	Notes
PMID-27352623	NIAT-Rituximab (n=37) vs. NIAT (n=38)	SAEs: 7 events (6 patients); Prostatitis (1, related to rituximab); No leukopenia observed	SAEs: 7 events (6 patients); Acute renal failure (2), Pleural effusion (1), Cancer (1)	0.87	Number of SAEs comparable; premedication prevented allergic reactions
PMID-31340979	Rituximab (n=28) vs. Control (n=27)	SAEs related to rituximab: 1/28 (0.03%)	SAEs: 1/27 (0.04%)	1	Minimal SAEs reported; no specific event details provided
PMID-33166580	Tacrolimus-Rituximab (n=43) vs. Corticosteroid-Cyclophosphamide (n=43)	SAEs: 6/43 patients; Serious AKI (1); AEs: AKI, hyperkalemia, diarrhea, tremor more common	SAEs: 11/43 patients; Serious infections (4/5); AEs: Leukopenia, Cushing syndrome more common	0.04 (AEs)	More AEs in comparator group; leukopenia linked to infections (P<0.0001)
PMID-33649098	Rituximab (n=37) vs. Cyclic Regimen (n=37)	SAEs: 8 events (7 patients, 19%); Infusion reactions (4), Cancer (2); AEs: Drug intolerance	SAEs: 6 events (5 patients, 14%); Leukopenia (3), Pneumonia (3), Cancer (1)	0.75 (SAEs)	Infusion reactions led to rituximab cessation; leukopenia/pneumonia more in cyclic arm
PMID-37688683	RTX/GC (n=35) vs. RTX (n=31)	AEs: 2/35 (5.7%); Fecal occult blood (1), COVID-19 (1)	AEs: 2/31 (6.5%); Upper respiratory infection (1), Allergic reaction (rash/dyspnea, 1)	1	No significant difference in AEs; COVID-19 impact on prognosis unclear

## 4 Discussion

Our meta-analysis highlights the significant therapeutic efficacy of low-dose, long-term rituximab (rituximab) regimens in the treatment of PLA2R-associated primary MGN. Notably, while many conventional treatment regimens demonstrate initial effectiveness, their therapeutic benefits often diminish over extended follow-up periods. In contrast, rituximab exhibits sustained efficacy, particularly in longer follow-up durations. Monthly administration of 100 mg rituximab has emerged as a potentially effective strategy for patients with low anti-PLA2R titers (14). This regimen is associated with not only comparable efficacy to recommended treatment protocols but also a significantly lower infection rate, making it particularly suitable for elderly patients (15). Furthermore, in a median follow-up period of 29 months, 65 patients achieved complete or partial remission, with a median remission time of 7.1 months. Remarkably, all 24 patients who were followed up for at least 4 years sustained remission (16).

The durability of rituximab’s therapeutic effects is underlined by a significant reduction in circulating CD19+ B cells from baseline to 24 months (P < 0.01) (17). However, disparities in cumulative remission rates between IMN and AMN groups were noted at the 12-month mark (65% vs. 90%, P = 0.045), suggesting that patient subgroups may exhibit differential responses to rituximab treatment (18). In another cohort, rituximab was administered biweekly, followed by a second course at 6 months for patients with persistent proteinuria despite B-cell recovery. Proteinuria was halved at 12 months, and among 14 patients completing follow-up, two achieved complete remission and six attained partial remission. Importantly, this protocol demonstrated minimal adverse effects (19).

Our meta-analysis underscores the significant therapeutic efficacy of low-dose, long-term rituximab regimens for PLA2R-associated primary MGN, particularly demonstrating sustained efficacy in extended follow-up. Monthly 100 mg rituximab is effective for patients with low anti-PLA2R titers, offering low infection rates suitable for elderly patients (14, 15). With a median 29-month follow-up, 65 patients achieved remission, and all over 4-year follow-ups maintained remission (16, 17). Patient subgroups may respond differently to rituximab (18). Rituximab plus low-dose tacrolimus (TAC) showed higher remission rates without increased adverse events (20, 21). Glomerular deposit clearance is slow, explaining persistent proteinuria post-rituximab. Our meta-analysis shows rituximab’s safety, but remission rates vary, suggesting patient-specific responses. Combination therapy (e.g., with cyclosporine) might accelerate remission in heavy proteinuria, but safety concerns exist. Further RCTs are needed to assess combination efficacy and safety, considering renal response time and long-term outcomes.

Our meta-analysis of rituximab combination therapies evaluated composite remission rates over a 12-month follow-up period. Two studies comprising 152 observations were analyzed. The common effect model yielded an odds ratio (OR) of 0.7902 (95% CI: 0.4082–1.5298, p = 0.4848), indicating no statistically significant difference in composite remission rates between rituximab-based regimens and alternative treatments. Similarly, the random effects model provided an OR of 0.9567 (95% CI: 0.0805–11.3650, p = 0.9720), reflecting considerable uncertainty in the effect estimate. High heterogeneity was observed (I² = 90.7%, p = 0.0010), underscoring variability across studies. Complete remission rates, however, reveal nuanced insights. In Ma et al., 2023, rituximab combined with glucocorticoids resulted in complete remission in 26 out of 31 patients (83.9%), compared to 60% in the rituximab group. Fernández-Juárez et al., 2021 (11) reported that rituximab combined with tacrolimus achieved complete remission in 51.2% of patients, compared to 79.1% in patients treated with corticosteroid and cyclophosphamide regimens. This significant heterogeneity, as indicated by the high I² value, necessitates a thorough exploration of potential sources to better understand the variability in treatment outcomes. Several factors may contribute to this observed variability across the included studies, including: patient demographics, where studies encompassed varying degrees of disease severity from severe MGN to PLA2R1-related and primary MGN, exhibited diverse age ranges with some including older populations, and demonstrated varied male to female participant ratios, all of which could significantly influence treatment responses; rituximab dosing and regimens, where studies employed different dosing protocols including standard and high-dose regimens, combined rituximab with various immunosuppressive agents like glucocorticoids, tacrolimus, and cyclophosphamide, and differed in the duration and intensity of combination therapies; study design and methodology, where despite all studies being RCTs, variations in inclusion/exclusion criteria, follow-up periods, and outcome measures, along with one study not reporting trial registration, and the geographical diversity of studies across France, USA, Spain, Italy, and China potentially introducing heterogeneity due to differing clinical practices and patient populations; and disease characteristics, where studies included patients with different MGN etiologies, such as PLA2R1-related, which could also contribute to the observed heterogeneity.

The phenomenon of spontaneous remission in a subset of patients with MGN poses a significant challenge in interpreting treatment efficacy, including the effects of rituximab. Approximately one-third of MGN patients experience spontaneous remission, necessitating an observational period of at least six months before initiating immunosuppressive therapy. This inherent variability in disease course can confound the assessment of treatment effects, particularly in studies with shorter follow-up periods. In our meta-analysis, the potential impact of spontaneous remission must be considered. For example, the patients with anti-CysR–restricted activity (nonspreaders) had higher rates of spontaneous remission. This suggests that patient selection based on specific PLA2R1 epitopes could influence observed remission rates, potentially introducing heterogeneity into our pooled results. Furthermore, the study by Debiec et al. acknowledged the possibility of bias due to high rates of late spontaneous remissions, emphasizing the need for RCTs to accurately assess rituximab’s efficacy. The authors also highlighted the ethical considerations of maintaining patients on NIAT (non-immunosuppressive alternative therapy) for extended periods, given the potential for disease progression. In light of these considerations, the observed remission rates in our meta-analysis may be influenced by the inclusion of patients who experienced spontaneous remission, particularly in studies with longer follow-up durations. While the randomized design of the included trials aimed to minimize this bias, the inherent variability in disease course and patient selection criteria across studies could still affect our findings. Future studies should aim to stratify patients based on their likelihood of spontaneous remission (e.g., epitope spreading, baseline proteinuria levels) to better assess the true efficacy of rituximab in this patient population.

Our meta-analysis underscores the significant efficacy of rituximab in treating PLA2R-associated primary MGN, with particular focus on the role of immune modulation in achieving remission. The therapeutic mechanisms of rituximab, a B-cell-depleting agent, are closely tied to changes in key immune markers, particularly in the context of PLA2R antibody (PLA2R-Ab) levels and B-cell depletion. Research has shown that patients who achieve remission with rituximab treatment have significantly lower PLA2R-Ab levels at 12 months compared to those who do not achieve remission (17.8 ± 21.2 RU/mL vs. 311.7 ± 356.0, P = 0.01) (22). This finding highlights the role of PLA2R-Ab as both a biomarker of disease activity and a predictor of treatment response. The sustained decrease in PLA2R-Ab levels may reflect the resolution of immune-mediated damage in MGN, correlating with clinical remission. This suggests that monitoring PLA2R-Ab levels can be an effective strategy for assessing the efficacy ofRituximab and predicting long-term treatment outcomes. In addition to PLA2R-Ab dynamics, rituximab’s effects on B-cell depletion play a crucial role in mediating clinical responses. At the 6-month and 12-month follow-ups, B-cell depletion was observed in a significant proportion of patients, with 84.3% of patients showing sustained B-cell depletion at 6 months (23). However, this depletion did not correlate strongly with clinical remission, as the odds ratio (OR) for depletion at 12 months was not statistically significant (OR = 2.25, 95% CI = 0.18–27.7, P = 0.66), indicating that while B-cell depletion is an important aspect of rituximab’s mechanism, it may not be the sole determinant of remission.

Further investigation into T-cell responses provides additional insights into rituximab’s immune-modulating effects. In the T-cell compartment, a significant increase in regulatory T cells (Tregs) was observed following B-cell depletion. Treg levels were found to increase 10-fold from baseline (1.2 ± 0.6% to 5.8 ± 0.7%, P = 0.02) at 12 months. Notably, this increase in Tregs was sustained only in the complete or partial remission (CR + PR) group and did not occur in non-responders (NR), suggesting that Treg expansion may play a role in promoting immune tolerance and disease resolution in responsive patients. This finding highlights the potential immunoregulatory role of Tregs in mediating rituximab efficacy in MGN. In contrast, activated T lymphocytes, specifically HLA-DR+CD8+ cells, showed a significant decrease afterRituximab therapy, with levels declining from 6 ± 1.1% at baseline to 1.5 ± 1.4% at 12 months (P = 0.05) (24). The reduction in activated T cells may reflect a shift towards immune homeostasis following B-cell depletion, further supporting the therapeutic potential of rituximab in modulating the immune response in MGN. In line with these immune mechanism observations, rituximab’s ability to target B cells and modulate T-cell responses may explain its effectiveness in MGN, particularly in patients with high PLA2R-Ab titers. The combination of B-cell depletion, Treg expansion, and reduced T-cell activation may contribute toRituximab’s sustained clinical efficacy, as evidenced by the favorable outcomes in long-term follow-up studies. Moreover, the specificity of PLA2R autoantibodies in MGN offers an additional layer of therapeutic insight. Anti-B-cell therapies like rituximab, through their ability to reduce PLA2R-Ab levels, not only offer a promising treatment option for MGN but also suggest that PLA2R-Ab measurement could serve as an important biomarker for tracking disease progression and predicting therapeutic response (25).

The studies included in this meta-analysis provide valuable insights into the role of rituximab in managing MGN and its potential to slow disease progression towards end-stage renal disease (ESRD). Conventional treatments like corticosteroid-cyclophosphamide regimens, while effective in preventing ESRD as per KDIGO guidelines, are associated with significant adverse events. In their study, only one patient, from the corticosteroid-cyclophosphamide group, progressed to ESRD, underscoring the efficacy of immunosuppressive therapy but also the risk of toxicity. Notably, this patient subsequently received rituximab without response, suggesting that rituximab may be more effective in earlier stages of disease or as a first-line treatment. Furthermore, the study by Fervenza et al. compared rituximab directly with cyclosporine. In this trial, ESRD developed in only one patient, who was in the cyclosporine group, and no patients in the rituximab group progressed to ESRD. This finding suggests that rituximab may offer comparable or even superior renal protection with a potentially more favorable safety profile, as indicated by the lower incidence of serious adverse events compared to cyclosporine. The observation that rituximab, in this study, demonstrated a trend towards preventing ESRD aligns with the hypothesis that targeted B-cell depletion can effectively modulate the immunopathogenesis of MGN, thereby preserving renal function. While these findings suggest a promising role for rituximab in slowing the progression of MGN and reducing the risk of ESRD, it’s crucial to acknowledge the limitations of the current data. The small number of ESRD events observed across these studies makes it challenging to draw definitive conclusions about the long-term renoprotective effects of rituximab. Future studies with larger sample sizes and longer follow-up periods are needed to further elucidate the impact of rituximab on renal survival in patients with MGN. Additionally, comparative studies with other immunosuppressive agents and evaluations of optimal dosing strategies are warranted to optimize treatment outcomes and minimize the risk of ESRD.

The included studies highlight the significant impact of rituximab on B-cell depletion in the treatment of MGN, with implications for both efficacy and potential long-term complications. It was observed that a higher dose rituximab protocol led to more effective B-cell depletion, correlating with improved remission rates. This underscores the importance of dosing strategies in achieving optimal therapeutic outcomes. However, the study by Fervenza et al. noted that CD19+ B-cell counts remained low at 12 months post-treatment, raising concerns about potential long-lasting immunosuppression and the risk of infections. While the authors suggested that this persistent depletion might indicate a prolonged therapeutic effect, they also acknowledged the absence of a direct correlation between B-cell counts at 12 months and proteinuria response in their previous studies, indicating the complexity of interpreting B-cell depletion in relation to clinical outcomes. Furthermore, the study noted that rituximab, while selectively depleting B-cells, carries the risk of hypogammaglobulinemia, a potential long-term complication that necessitates careful monitoring and management. Thus, while rituximab demonstrates efficacy in MGN by targeting B-cells, the delicate balance between achieving therapeutic B-cell depletion and mitigating the risks of prolonged immunosuppression and hypogammaglobulinemia requires careful consideration in clinical practice.

While this meta-analysis offers valuable insights into the efficacy of rituximab in treating MGN, several limitations should be considered. The studies included in this analysis exhibited moderate to high heterogeneity, particularly for the 12-month and 24-month follow-up periods, which may limit the generalizability of the findings. Most of the studies included were observational in nature and had relatively small sample sizes, which could lead to bias in the reporting of outcomes. Additionally, the lack of comparator groups in some studies may have further contributed to this bias. Differences in treatment protocols, such as the use of rituximab in combination with other therapies or varying dosages, could introduce variability in the treatment outcomes, complicating the interpretation of the pooled results. A few studies reported incomplete follow-up data, particularly at the longer follow-up periods (12 months and 24 months), which could impact the accuracy of the remission rates. Furthermore, we must acknowledge the potential for publication bias, as positive results are more likely to be published. Due to the limited number of included studies, formal assessment of publication bias using funnel plots or Egger’s test was not feasible. The variability in follow-up duration across studies may also influence the assessment of efficacy and safety, as shorter follow-up periods may not capture delayed adverse events or long-term effects. Additionally, our analysis primarily relies on short-term data, and the lack of long-term safety data beyond 24 months limits our ability to fully evaluate the long-term safety profile of rituximab. Therefore, potential risks associated with long-term use require further investigation. However, our findings, in conjunction with current clinical guidelines, suggest that rituximab may be a promising first-line treatment option for patients with primary MGN, potentially replacing traditional therapies such as cyclophosphamide and steroids (26). This is particularly relevant in the post-PLA2R era, where autoantibodies to PLA2R and THSD7A have become essential diagnostic and monitoring tools, allowing for more targeted and personalized treatment strategies (27). The clinical remission rates observed following first-line rituximab therapy in our analysis were notably higher than those reported for second-line rituximab therapy, emphasizing the importance of early intervention. Moreover, the evolving understanding of antigenic targets in MGN, beyond PLA2R and THSD7A, could introduce selection bias in our analysis. As included studies didn’t report on newer targets like NELL1, EXT1/2, NCAM1, and Semaphorin 3B, we cannot quantify this bias. Future research should explore rituximab’s efficacy across varying antigenic profiles.

Despite these limitations, this meta-analysis also possesses several notable strengths. By focusing exclusively on RCTs, we aimed to provide a rigorous and unbiased assessment of rituximab’s efficacy and safety in MGN. This selection criterion enhances the internal validity of our findings, minimizing the impact of confounding variables inherent in observational studies. Furthermore, the comprehensive search strategy employed, encompassing multiple databases and adhering to established guidelines, aimed to maximize the retrieval of relevant studies, thereby reducing the risk of selection bias. The inclusion of studies with varied patient demographics and clinical presentations, while contributing to heterogeneity, also enhances the external validity of our findings, potentially broadening the applicability of our conclusions to a wider patient population. Additionally, our analysis provides a consolidated view of rituximab’s impact on key clinical outcomes, offering clinicians a valuable resource for evidence-based decision-making in the management of MGN.

## 5 Conclusions

This meta-analysis suggests that rituximab may have a positive effect on achieving complete and composite remission in patients with MGN, particularly over a 24-month follow-up period. Although the results were not statistically significant for complete remission at shorter follow-up intervals (6 and 12 months), there was an observed trend towards improved outcomes in patients treated with rituximab. The findings also emphasize the need for more well-designed, large-scale, RCT to confirm the efficacy of rituximab and to establish optimal treatment protocols. Additionally, further research is needed to explore the long-term effects and adverse event profile of rituximab in this patient population.

## Data Availability

The original contributions presented in the study are included in the article/supplementary material. Further inquiries can be directed to the corresponding author.
